# Retrospective Evaluation on the Use of a New Polysaccharide Complex in Managing Paediatric Type 1 Diabetes with Metabolic Syndrome (MetS)

**DOI:** 10.3390/nu13103517

**Published:** 2021-10-07

**Authors:** Stefano Stagi, Valeria Papacciuoli, Daniele Ciofi, Barbara Piccini, Giovanni Farello, Sonia Toni, Marta Ferrari, Francesco Chiarelli

**Affiliations:** 1 Department of Health Sciences, University of Florence, Anna Meyer Children’s University Hospital, 50139 Florence, Italy; daniele.ciofi@meyer.it (D.C.); marta.ferrari@unifi.it (M.F.); 2 Pediatric Diabetology Unit, Anna Meyer Children’s University Hospital, 50139 Florence, Italy; valeria.papacciuoli@gmail.com (V.P.); barbara.piccini@meyer.it (B.P.); sonia.toni@meyer.it (S.T.); 3 Department of Paediatrics, University of L’Aquila, 67100 L’Aquila, Italy; giovanni.farello@univaq.it; 4 Department of Paediatrics, University of Chieti, 66100 Chieti, Italy; chiarelli@unich.it

**Keywords:** children, Policaptil Gel Retard^®^, type 1 diabetes, obesity, overweight, haemoglobin A1c, treatment, metabolic syndrome, MetS, glycaemic index, insulin dosing

## Abstract

Background: Children and adolescents affected by type 1 diabetes have an increased risk of being overweight or obese and of suffering from cardiometabolic symptoms. Aims: To retrospectively evaluate the effects of a new complex of polysaccharide macromolecules, Policaptil Gel Retard^®^ (PGR), on auxological and metabolic parameters, glycaemic variability and control parameters in paediatric patients with type 1 diabetes and metabolic syndrome (MetS). Patients and Methods: Data for 27 paediatric patients with a diagnosis of type 1 diabetes in conjunction with obesity and MetS of at least 5 years’ standing were collected and retrospectively studied. Of these, 16 (median age 12.9, range 9.5–15.8 years) had been adjunctively treated with PGR and 11 (median age 12.6, range 9.4–15.6 years) had not been treated with PGR. Auxological, metabolic and glycaemic control and variability parameters and insulin dosing were compared after 6 months in the two groups. Results: PGR significantly reduced BMI standard deviation score (SDS) (*p* < 0.005), waist SDS (*p* < 0.005), HbA1c (*p* < 0.05) and daily mean insulin dose requirement (*p* < 0.005). A significant improvement was also observed in the metabolic and glycaemic variability parameters of mean daily blood glucose (BG) levels (*p* < 0.005), SD of daily BG levels (*p* < 0.0001), mean coefficient of variation (*p* < 0.05), LBGI (*p* < 0.0001), HBGI (*p* < 0.0001), J-index (*p* < 0.005), total cholesterol (*p* < 0.005), HDL-cholesterol (*p* < 0.005) and LDL-cholesterol (*p* < 0.005) and triglycerides (*p* < 0.05). Conclusions: PGR produces a good auxological and metabolic response in obese patients with MetS who are affected by type 1 diabetes. It led to a significant reduction in BMI SDS, waist SDS and an improvement in glucose control and variability as well as in other MetS parameters. The use of polysaccharide compounds, especially if associated with appropriate dietary changes, may help achieve treatment targets in type 1 diabetes and reduce the risk that patients develop metabolic syndrome.

## 1. Introduction

Diabetes mellitus is the most common endocrine disorder among children and adolescents [1]. It is caused by impaired insulin secretion and/or action and is characterised by chronic hyperglycaemia affecting the metabolism of carbohydrates, fats and proteins [1,2]. Type 1 diabetes is an immune-mediated disorder characterised by T cell-mediated autoimmune destruction of the β cells of the pancreas, leading to a deficit or absence of insulin [3].

In treating type 1 diabetes, the main objective is glycaemic control to prevent hypoglycaemic episodes as well as long-term hyperglycaemia-related complications [4,5].

This is particularly important considering the high morbidity and mortality of the disorder. Often, patients have a reduced quality of life due to long-term microvascular and neurological complications [6]. The risk of developing complications seems to be more closely related to glycaemic variability than the presence of hyperglycaemia [4,5,6].

For patients with type 1 diabetes, the administration of insulin, dietary controls and regular physical activity can achieve “near normoglycaemia” [6,7,8]. Medical nutrition therapy is important for managing type 1 diabetes as the quantity and type of carbohydrates eaten significantly affects postprandial blood glucose levels [9]. According to the American Diabetes Association (ADA), treatments centred on glycaemic index (GI) and glycaemic load (GL) can lead to modest additional benefits [9,10,11,12]. Results from various randomised clinical trials suggest that low-GI diets may [10,13,14,15,16,17] or may not [18,19] reduce glycaemic response in diabetic subjects. Given these data, in order to obtain the most positive treatment outcomes, the diet of these patients should consist principally of low-GI foods [12].

There is much evidence to demonstrate that type 1 diabetes patients, like patients with type 2 diabetes, are at a greater risk than the general population of being overweight or obese, of developing metabolic syndrome (MetS) and of developing resistance to injected insulin [20,21,22,23,24,25,26].

The prevalence of MetS in children, according to a metanalysis published in 2013, is around 3% [27]. However, few studies have focused on the prevalence and characteristics of MetS in paediatric patients with type 1 diabetes, although some data suggest that 9.5–15.0% of type 1 diabetes patients may present this disorder [28,29].

In a previous study, we tested the effect of Policaptil Gel Retard^®^ (PGR), a polysaccharide macromolecule complex in obese children and adolescents affected by severe hyperinsulinism and insulin resistance [30]. In a second study on patients affected by obesity and MetS, the complex was administered in association with metformin [31]. Reductions in peak blood glucose and insulin levels were observed in both studies. 

Interestingly, a recent study by Fornari et al. on obese children showed that the intake of PGR is associated with a significant reduction in appetite, ghrelin and triglycerides in the postprandial period [32].

The aim of this study was to establish the effect of this complex on glycaemic variability, insulin sensitivity and insulin dosing in obese children and adolescents with type 1 diabetes and MetS.

## 2. Subjects and Methods

This was a 6-month retrospective observational study involving data for twenty-seven (14 males, 13 females; median age 12.8, range 9.4–15.8 years) Caucasian patients with type 1 diabetes who were followed up with by our unit between January 2011 and January 2019. The patients lived in 3 different areas of Italy and were evaluated and monitored for weight gain and MetS. 

Type 1 diabetes was diagnosed on the basis of fasting plasma glucose (FPG) levels ≥ 126 mg/dL (7.0 mmol/L) or 2 h postprandial glucose levels ≥ 200 mg/dL (11.1 mmol/L) [33,34], in addition to the sudden onset of symptoms such as polyuria, polydipsia, unexplained weight loss and/or diabetic ketoacidosis, fasting C-peptide assay < 0.3 pmol/mL and stimulated C-peptide < 0.6 pmol/mL, positive pancreatic autoantibodies (GAD-65, IAA and ICA) and the continued need for insulin injections since the time of diagnosis [35].

The inclusion criteria of the study were a diagnosis of type 1 diabetes of least 5 years’ standing, an age between 8 and 18 years, the presence of MetS and obesity and an insulin dose of >0.8 u/kg/day at the time of the study. The exclusion criteria were an age <8 or >18 years, BMI > 95th percentile at type 1 diabetes diagnosis, type of diabetes other than type 1 diabetes, existing chromosomal and chronic diseases (thyroid, malabsorption and/or gastrointestinal disorders), chronic renal insufficiency, coexisting infections, cancer and use of medications such as topical or systemic glucocorticoids, anticonvulsant therapy, lipid-lowering medication, oral hypoglycaemic agents and sexual steroids. The exclusion criteria also included dietary restrictions other than those in keeping with the design of the study.

The study was conducted according to the Declaration of Helsinki and European Guidelines on Good Clinical Practice. Ethical approval was obtained from the Meyer Children’s University Hospital Ethics Committee (number 145/2015). Written informed consent was obtained from the parents of the retrospectively enrolled patients after they had acknowledged their full understanding of the objectives of the research.

### 2.1. Study Design 

As per clinical practice in our hospitals, type 1 diabetes patients with obesity and MetS were evaluated frequently through clinical and laboratory examinations. 

The collection of medical histories and physical examinations were carried out by qualified practitioners who collected the following information: age, gender, duration of type 1 diabetes (years), total daily insulin requirements (U/kg/day), fasting, pre- and postprandial glucose values, HbA1c levels, physical activity (hours/week) and presence of diabetes gravidarum during mother’s pregnancy. Family histories of cardiovascular diseases, hypertension, hypercholesterolemia, obesity and diabetes (type 1, type 2, gestational or other diabetes) were recorded for first- and second-degree family members. Nutrient intake was also recorded for the whole sample using medical charts and standard interviews [30]. At each visit, weight, height, BMI, pubertal staging and systolic (SBP) and diastolic (DBP) blood pressure were recorded. 

The patients’ blood was tested for HbA1c levels and lipid profiles (triglycerides [TG], total cholesterol [TC], low-density lipoprotein [LDL] cholesterol and high-density lipoprotein [HDL] cholesterol) in a state of metabolic stability after a fast of at least 8 h. Patients were also tested to provide a renal profile and full blood count and for IgA anti-tissue transglutaminase antibody (tTG) and total IgA.

The following parameters regarding glycaemic variability were investigated on the basis of medical records and interviews (see Supplementary Materials): frequency of hypoglycaemic (glucose meter readout < 3.88 mmol/L) and hyperglycaemic (glucose meter readout > 10.00 mmol/L) episodes, percentage of readings in the target range (between 4.44 and 7.77 mmol/L), the number of episodes of ketosis and ketonemia, the mean number of insulin boluses, mean insulin dose per day, mean number of daily glucose determinations, mean daily glucose values (on average, one measurement before each meal (breakfast, lunch and dinner), one measurement 2 h after each meal (breakfast, lunch and dinner) and one measurement at 3:00 a.m.) and their SDs, mean coefficient of variation, Average Daily Risk Range (ADRR: a sensitive measure of glycaemic variability (GV)), Low or High Blood Glucose (BG) Index (LBGI and HBGI provide early risk indicators for hypoglycaemia and hyperglycaemia), Glycaemic Risk Assessment Diabetes Equation (GRADE), J-index and Mean Amplitude of Glycaemic Excursion (MAGE) and Mean Daily Differences (MODD) in patients for whom the necessary information was available. Calculations were performed also by EasyGV calculator (Nuffield Department of Primary Care Health Sciences, Medical Sciences Division, Oxford University, UK). 

As all children with type 1 diabetes have hyperglycaemia, the patients were classified as having MetS if they presented two or more of the following: BMI above the 97th percentile (and waist circumference ≥ 90th percentile), triglyceride levels above the 95th percentile, HDL cholesterol levels below the 5th percentile and elevated blood pressure, defined as systolic and/or diastolic blood pressure above the 95th percentile for age and sex [23].

### 2.2. Study Protocol

In analysing the data, we divided the patients into two groups, according to whether they had received PGR treatment or not: sixteen patients (group 1: 8 males, 8 females, mean age at study onset 12.9, range 9.5–15.8 years) had been treated with PGR, whereas eleven patients (group 2: 6 males, 5 females, mean age 12.6, range 9.4–15.6 years) had not been treated with PGR and acted as controls.

All patients were evaluated every three months: at baseline (T_0_) and after 3 (T_1_) and 6 months (T_2_). For the treated group at T_0_, as per clinical practice, before the treatment with PGR, all patients were given written instructions and were asked to take 3 tablets of PGR before their two main meals. Parents were asked to complete a written questionnaire at the follow-ups, and occasionally interviewed by email and telephone in order to evaluate their children’s compliance, while bottle counts were periodically performed.

PGR is the Active Pharmaceutical Ingredient (API) of Libramed tablets (Aboca Spa Company, Sansepolcro, Arezzo, Italy), able to slow the rate of carbohydrate absorption, thereby decreasing glycaemic and insulinemic peak intensity [30]. The complex contains polysaccharide macromolecules (cellulose, hemicellulose, pectin, mucilage) and is derived from the following high-fibre raw materials: glucomannan (*Amorphophallus konjac*), cellulose, Opuntia pulp stem (*Opuntia ficus indica*), chicory root (*Cichorium intybus*), freeze-dried mallow root mucilage (*Althaea officinalis*), freeze-dried flaxseed mucilage (*Linum usitatissimum L*) and freeze-dried linden flower mucilage (*Tilia platyphyllos Scop*) [30]. 

All patients were instructed to obtain 6–10 points of self-monitored BG values (SMBG). The ideal BG level for the M value in this study was set at 5.5 mmol/L, with 4.44 mmol/L as the lower limit and 7.77 as the upper limit of the target range. GV was defined as the degree to which a patient’s BG fluctuated between high and low levels [36]. As reported above, glucose variability was determined by several SMBG-derived indices, each sensitive to a different aspect of variability (see Supplementary Materials for the explication) [29,37,38,39,40,41,42,43,44]. In addition, the frequencies of hypoglycaemic and hyperglycaemic events (glucose meter readout of <3.88 and >10 mmol/L, respectively) were assessed using standard patient diaries [38].

As per clinical practice, to exclude changes in diet and physical activity during the study, habitual food consumption and hours of screen time (computer, TV and video) and physical activity per week were measured with a quantitative food frequency questionnaire (QFFQ) and physical activity questionnaire, as previously reported [30]. We analysed dietary records for fat (total, saturated, monounsaturated and polyunsaturated), cholesterol, protein, carbohydrate, fibre and calories. GIs were used as previously reported [30]. We determined GL by multiplying the carbohydrate content (in g) of each food consumed by an individual over one day by the food’s GI [30]. GI is a parameter for classifying foods according to postprandial glycaemic response [11], while GL reflects the glycaemic values obtained after consuming a meal containing varying quantities of carbohydrates [12]. 

All patients were under treatment with insulin injections 4 times/day. These included three mealtime doses of rapid-acting analogues (fixed doses of insulin for food and adjusting doses according to blood glucose levels) and one dose of insulin glargine (Sanofi, Paris, France) before bedtime (9:00 or 10:00 p.m.).

Nutritional status was classified according to BMI: patients with a BMI ≥95th percentile for age and gender were considered obese, according to the percentiles designed by Cacciari et al. [45]. 

We determined IR using formulas validated by the hyperinsulinemic–euglycemic clamp technique. The first formula, estimating glucose disposal rate (eGDR), allows the glucose release rate to be estimated [46] and is calculated by an equation involving the following clinical and laboratory parameters: eGDR = 24.4 − (12.97 × W/H) − (3.39 × AH) − (0.60 × A1c); where W/H is the waist/hip ratio and AH indicates the presence of arterial hypertension (yes = 1, no = 0). The second formula estimates the insulin sensitivity score (ISS) [46] and is calculated as: LogeIS = 4.64725 − 0.02032 (W, cm) − 0.0977 (A1c, %) − 0.00235 (TG, mg/dL), where LogeIS is the logarithm of IS, W is the waist size and TG is the triglyceride level [47,48]. The total dose of insulin per day was reported for each patient. We also assessed insulin sensitivity by comparing insulin dose and weight (as proposed by Reinehr et al.) [26]. We considered insulin sensitivity (<1.0 U/kg/d), mild insulin resistance (≥1.0–<1.8 U/kg/d) and severe insulin resistance (≥1.8 U/kg/d).

With regard to MetS criteria [23], we defined dyslipidaemia as the presence of one or more abnormal serum lipid concentration [49]. The cut-off points for abnormal lipid levels (TC ≥ 5.17 mmol/L, LDL cholesterol ≥ 3.36 mmol/L, HDL cholesterol ≤ 1.03 mmol/L and TG ≥ 1.70 mmol/L) were those used by the American Diabetes Association [49]. 

### 2.3. Auxological and Clinical Methods

Height was measured, in triplicate, to the nearest 0.5 cm using a wall-mounted stadiometer and weight was measured by a standard physician’s beam scale to the nearest 0.1 kg; patients were weighed without shoes and dressed in light underwear. The same trained practitioners made the measurements. Waist circumference was measured to the nearest 0.1 cm at the end of normal expiration using a non-elastic tape measure placed midway between the lowest rib margin and the iliac crest [50,51]. The coefficient of variation (CV) values were <1%. 

Body mass index (BMI) was calculated as weight in kilograms divided by height in metres squared (kg/m^2^). Age-related reference values for height, weight and BMI were obtained from specific charts reflecting standards for the Italian paediatric population [45].

Height, weight, BMI, waist circumference and hip circumference were normalised for chronological age and converted to standard deviation scores (SDS) [52]. The following formula was used: (patient value − mean of age-related reference value)/standard deviation of the age-related reference value. Tanner and Whitehouse’s criteria, with an orchidometer for boys, were used for pubertal staging [53]. 

Trained personnel measured blood pressure three times by auscultation with a mercury sphygmomanometer on the right arm of the patient, who had been sitting quietly for 5 min with the back supported, feet on the floor, right arm supported and cubital fossa at heart level [54]. Practitioners used an appropriate cuff size. The 5th Korotkoff sound was taken for diastolic blood pressure categorisation. Mean systolic and diastolic values were recorded and stratified according to the paediatric percentiles of the National High Blood Pressure Education Program Working Group on High Blood Pressure in Children and Adolescents [54]. We also converted mean values into SDS to aid statistical analyses [54].

### 2.4. Laboratory Methods

All participants were examined in the morning after overnight fasting. Serum and plasma were immediately separated and stored at −20 °C in multiple vials for later analysis. 

Serum glucose (Dimension RXL system, Dade Behring, Dallas, TX, USA) and serum insulin (IMMULITE 2000 analyser, Siemens Healthcare Diagnostics, Marburg, Germany) levels were measured using immunoenzymatic assays, and glycosylated haemoglobin (HbA1c) levels were determined using high-performance liquid chromatography (DIAMAT, Bio-Rad, Richmond, CA, USA). The normal range for HbA1c was 4.2–6.0%, and the CV at 5.5% was 4.8.

Home blood glucose measurements were performed using the Roche Diagnostics Accu-Chek Aviva Nano^®^ glucose meter. We transferred logged data to a computer utilising the Roche Diagnostics Accu-Chek Smart Pix^®^ device (Roche Diabetes Care Italy S.p.A., Monza, Milan, Italy). 

Ketonuria was measured using Bayer Ketostix^®^ urine strips and ketonemia was determined by Abbott MediSense Optium Xceed Meter^®^.

Total cholesterol, HDL cholesterol and triglycerides (TG) were measured using routine laboratory methods. Low-density lipoprotein (LDL) cholesterol was calculated using the Friedwald formula: LDL = total cholesterol − HDL cholesterol − TG/2.2 [30].

### 2.5. Statistical Analysis

Data analysis was performed with the use of “Statistical Package for the Social Sciences for Windows” (SPSS, Inc, Chicago, IL, USA), version 13.0. Descriptive statistics are presented as numbers (percentages), median and range and mean ± SD values. Histograms and Shapiro–Wilk tests were used to verify the normality of continuous data. The variation of continuous variables at the beginning and end of observation was analysed by paired Student *t*-tests for parametric data, while the Wilcoxon signed-rank test was used for nonparametric analysis. Categorical variables were compared by the χ2 test or Fisher’s exact test. All tests were two-sided, and values of *p* < 0.05 were considered statistically significant.

## 3. Results

**Figure 1:**
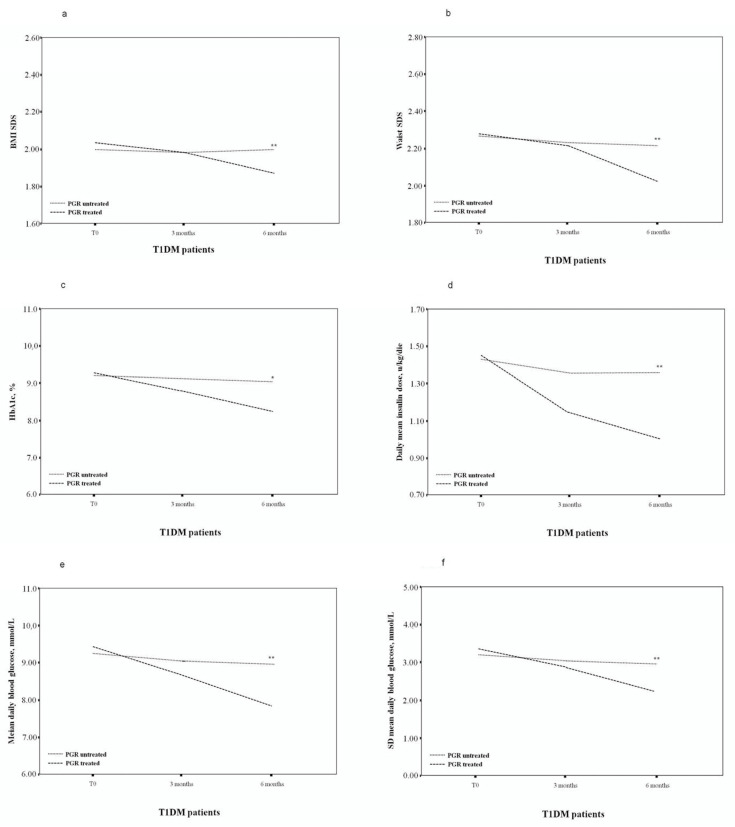
Body mass index (BMI) standard deviation score (SDS) (**a**), waist SDS (**b**), HbA1c, % (**c**), daily median insulin dose (**d**), mean daily BG levels (**e**), SD of the daily BG levels (**f**) of PGR-treated and untreated patients. * *p* < 0.05; ** *p* < 0.005.

Clinical, laboratory and demographic data are summarised in Table 1 and Table 2 and Figure 1. Glucose variability and hypo- or hyperglycaemic measurements are also included in Table 2. 

### 3.1. Overall Baseline Data

Among our type 1 diabetes patients affected by obesity and MetS, we observed a high prevalence of type 2 diabetes (group 1: 56.2%; group 2: 63.6%) and obesity (group 1: 18.7%; group 2: 18.1%) in family members (Table 1). Approximately 62.5% of group 1 and 54.5% of group 2 came from southern Italy where type 2 diabetes and obesity are more common than in the other regions of Italy. Two patients (two girls, 13.1 and 12.2 years) had reduced (albeit sufficient) self-monitoring BG data. At baseline, the mean insulin dose for group 1 was 1.47 ± 0.49 U/kg/day (1.00–1.99 U/kg/day) and for group 2, 1.45 ± 0.43 U/kg/day (1.01–1.97 U/kg/day), with no difference between the two groups and between females and males. Twenty patients (74.1%; group 1: 13 patients, 76.5%; group 2: eight patients, 72.7%) were classified as mildly insulin resistant and seven patients (25.9%; group 1: four patients, 23.5%; group 2: three patients, 27.3%) as severely insulin resistant. 

At baseline, we did not observe differences in the degree of obesity (BMI SDS and waist SDS), GI, GL or physical activity among these groups (Table 1 and Table 2). However, the severely insulin-resistant patients had been diagnosed for a longer time (7.2 ± 0.5 years) than the mildly insulin-resistant group (6.3 ± 0.6 years, *p* < 0.005). Patients with severe insulin resistance also had higher HbA1c levels (9.5 ± 0.4 vs. 8.9 ± 0.5, *p* < 0.005). Fatty liver disease was found in 17 (62.9%) patients. All patients had dyslipidaemia. Hypercholesterolaemia was found in 14 patients (51.8%), low HDL-cholesterol in 17 (62.9%), high LDL-cholesterol in 18 (66.6%) and high triglycerides in 15 (55.5%). Finally, high SBP levels were found in 13 patients (48.1%) and high DBP in 11 (40.7%) (Table 2). 

The patients experienced high glycaemic variability, as the mean coefficient of variation was 36.39%. Additionally, 11.1% of patients experienced hypoglycaemia in the morning and 22.2% at night. The number of hypoglycaemia episodes was 4.0 ± 1.2 in the morning and 6.7 ± 3.1 at night (Table 2).

### 3.2. Group 1 and 2 Baseline Data (T_0_)

At baseline, we did not observe differences in the height SDS, in the degree of obesity (BMI SDS and waist SDS), prepubertal/pubertal ratio, GI, GL screen time or physical activity between the two groups (Table 1 and Table 2). These patients did not present differences also in metabolic parameters, the parameters of glycaemic control (HbA1c, mean daily BG, mean fasting BG, mean pre-lunch BG, mean pre-dinner BG, mean postprandial BG, mean coefficient of variation), the mean insulin dose or the main parameters used for the variability evaluation (LBGI, HBGI, GRADE, J-index, MODD, ADRR, hypoglycaemia index, MAGE, hyperglycaemia index, index of Glycaemic Control, nocturnal and morning hypoglycaemia episodes per month, etc.) (Table 1 and Table 2).

### 3.3. Group 1 and 2 T_1_ vs. T_2_ Data

After 6 months of PGR treatment, the group 1 patients presented a significantly reduced BMI SDS (T_2_ 1.88 ± 0.16 vs. T_0_ 2.04 ± 0.12, *p* < 0.005), with a Δ BMI SDS of −0.16 ± 0.04, compared to the untreated patients (*p* < 0.05). In PGR-treated patients, a significant decrease in waist SDS (T_2_ 2.04 ± 0.19 vs. T_0_ 2.29 ± 0.20, *p* < 0.005), with a Δ waist SDS of −0.25 ± 0.01, was also reported.

HbA1c was significantly lower in PGR-treated patients: T_2_ 8.20% (7.0–9.5%) vs. T_0_ 9.30% (7.4–10.2%) (*p* < 0.05), also in comparison with group 2 patients (*p* < 0.05).

In patients treated with PGR, we also observed a significant decrease in daily mean insulin dose (T_2_ 1.03 ± 0.29 vs. T_0_ 1.47 ± 0.49 U/kg/day) (*p* < 0.005), which was already reduced after just 3 months of treatment (T_1_ 1.16 ± 0.34 vs. T_0_ 1.47 ± 0.49 U/kg/day, *p* < 0.05). Again, this was significantly different from untreated patients (*p* < 0.05). 

A significant improvement was shown in PGR-treated patients in metabolic parameters and glycaemic variability, with a significant decrease in mean daily BG levels (T_2_ 7.99 ± 1.12 vs. T_0_ 9.48 ± 1.57 mmol/L, *p* < 0.005), fasting BG levels (T_2_ 7.63 ± 0.88 vs. T_0_ 8.96 ± 1.31 mmol/L, *p* < 0.005), pre-lunch BG levels (T_2_ 9.21 ± 1.73 vs. T_0_ 11.42 ± 2.48 mmol/L, *p* < 0.05), pre-dinner BG levels (T_2_ 10.01 ± 2.07 vs. T_0_ 11.63 ± 2.26 mmol/L, *p* < 0.05) and postprandial BG levels (T_2_ 10.41 ± 2.02 vs. T_0_ 12.09 ± 2.21 mmol/L, *p* < 0.005) (Table 2). Improvement was evident in all these parameters in PGR-treated patients compared to untreated patients (*p* < 0.05).

Moreover, a reduction in the SD of daily BG levels (T_2_ 2.28 ± 0.43 vs. T_0_ 3.45 ± 0.57 mmol/L, *p* < 0.0001), mean CV levels (T_2_ 28.53% vs. T_0_ 36.39%, *p* < 0.05), LBGI levels (T_2_ 2.55 ± 1.87 vs. T_0_ 5.64 ± 2.33, *p* < 0.0001), HBGI levels (T_2_ 5.46 ± 1.91 vs. T_0_ 9.87 ± 2.79, *p* < 0.0001) and J-index levels (T_2_ 34.17 ± 9.11 vs. T_0_ 54.16 ± 17.22, *p* < 0.005) was observed (Table 2). The mean (SD) dietary GI and GL and the time spent weekly on exercise did not change significantly from the values at the beginning of the study (Table 2). Patients treated with PGR presented improvements in the SD of daily BG levels (*p* < 0.005), LBGI levels (*p* < 0.005), HBGI levels (*p* < 0.0001) and J-index levels (*p* < 0.005), in comparison with non-treated patients (Table 2).

Finally, there was a significant reduction in total cholesterol (T_2_ 4.81 ± 0.41 vs. T_0_ 5.41 ± 0.64, *p* < 0.005) and LDL-cholesterol (T_2_ 3.12 ± 0.37 vs. T_0_ 3.73 ± 0.52, *p* < 0.0005) and a significant increase in HDL-cholesterol (T_2_ 1.01 ± 0.13 vs. T_0_ 0.86 ± 0.13, *p* < 0.005), triglycerides (T_2_ 1.51 ± 0.31 vs. T_0_ 1.81 ± 0.39, *p* < 0.05), SBP and DBP values (*p* < 0.05).

### 3.4. Safety Data

The incidence of adverse events was 12.5% (two patients) over the 6 months of treatment; these were exclusively gastrointestinal events including diarrhoea (one patient, 6.2%), flatulence and abdominal pain (one patient; 6.20%). In no case was it necessary to cease treatment and no patient suffered from serious gastrointestinal complications, although one continued to report moderate gastrointestinal symptoms (flatulence and abdominal pain). 

## 4. Discussion

Our findings in children and adolescents with type 1 diabetes, obesity and MetS suggest that PGR significantly lowers BMI and improves adiposity parameters, supporting our previous work and the literature on severely obese children and adolescents with insulin resistance and a family history of obesity and type 2 diabetes [30,31,32].

Our data also suggest that the use of this compound may allow the daily mean insulin dose to be reduced and produce an improvement in glucose metabolism parameters, such as HbA1c values, BG mean values and BG variability parameters. Our results support the importance of medical nutrition therapy in managing existing type 1 diabetes [9], indicating that a low GI-diet or the use of GI-lowering supplements may be helpful. 

Given the increased global incidence of obesity and MetS in paediatric type 1 diabetes patients [55], in keeping with an increased general incidence of type 1 diabetes worldwide [56], our study could be important for managing these patients more effectively. A large study involving 500 children with type 1 diabetes showed that almost one third of European children were overweight or obese [57], whereas in another study, about one quarter of 451 Indian patients presented MetS [58]. Type 1 diabetes leads to higher cardiovascular risk [59] and higher rates of morbidity and mortality [60]. MetS, which indicates an elevated risk of diabetic complications [61], is significantly more frequent in obese subjects with type 1 diabetes [23]. Factors affecting weight gain include degree of glycaemic control [61], gender, intensity [62] and mode of insulin treatment (pump versus MDI) [58], the presence of comorbidities such as coeliac or thyroid disease [23], drug use and the presence of eating disorders [23]. Insulin therapy can cause weight gain due to an over-replacement of insulin which produces a general anabolic effect, decreased energy expenditure, greater carbohydrate intake in response to the perceived risk of hypoglycaemia and the non-physiological mode administrating insulin [23]. Being overweight increases insulin resistance, which can exaggerate the negative effects of treatment [22]. The use of this compound, possibly in association with a low GI/GL diet, could lead to an improvement in the parameters associated with MetS. More specifically, a recent study by Fornari et al. has shown that the intake of PGR is associated with a significant reduction in appetite, ghrelin and triglycerides in obese children, confirming the effect of PGR in affecting appetite, metabolic and hormonal postprandial profile [32]. Therefore, because of its characteristics, PGR could represent a promising treatment option in these patients. The effect of PGR in reducing mean insulin dosage and mean and pre- and post-meal BG and in improving overall glucose variability may be due to a reduction in postprandial glycaemic peaks with better blood glucose stability. We found a reduction in hypoglycaemic and hyperglycaemic episodes, but perhaps because of the limited number of cases included in our study, it was not significant and did not present a clear trend. Instead, there was a significant decrease in the number of nocturnal hypoglycaemic episodes following treatment with PGR. However, these results could also be related to physical activity and diet, which were not a focus of this study. 

Interestingly, we found a high frequency of type 2 diabetes and obesity in first- and second-degree relatives for type 1 diabetes patients with obesity and MetS. Similar findings have been reported in previous studies [25,62]. We hypothesise that intensive therapy leads to the expression of several components of the central obesity syndrome phenotype in a subset of type 1 diabetes patients with a positive family history for type 2 diabetes and/or obesity [63]. 

Interestingly, a recent study by Guarino G et al. [64] in adults demonstrated the non-inferiority of PGR compared to metformin on glycaemic control in obese adults with MetS and type 2 diabetes, and a superiority in terms of reductions in lipid values and tolerance, constituting new insights for the use of PGR in adult patients with type 2 diabetes and MetS.

We are aware of our study’s limitations, which include the small number of cases, a problem found in other papers studying paediatric type 1 diabetes patients [56], as well as the study’s retrospective nature, the lack of a control group and a lack of data on body fat. 

Moreover, although the results of this therapy are promising, currently, the literature is lacking studies that have investigated the correlation between PGR and parameters of glucose and lipid metabolism. This aspect is certainly a limitation for our work, even though the few studies available agree in defining the metabolic role of PGR in these categories of patients. 

Our results clearly point out the need for more clinical trials involving larger numbers of patients which, if our results are confirmed, may be able to establish to what extent and by what means PGR could ameliorate the development of type 1 diabetes complications. 

## 5. Conclusion

Type 1 diabetes patients with MetS showed a good auxological and metabolic response to PGR. There were significant decreases in BMI and waist SDS and an improvement in glucose control and variability, as well as many MetS parameters. The use of such polysaccharide compounds, especially if associated with appropriate dietary changes, may be useful in achieving treatment targets in type 1 diabetes, thus reducing the risk that a patient develops metabolic syndrome.

## Figures and Tables

**Table 1:** Baseline demographic and clinical characteristics in Policaptil Gel Retard^®^ (PGR)-treated and untreated children with type 1 diabetes.

Variable	Characteristics		
	Treated	Untreated	*p*
Subjects, number (M:F)	16 (8/8)	11 (6/5)	-
Age, years (median and range)	12.9 (9.5–15.8)	12.6 (9.4–15.6)	-
Prepubertal/pubertal ratio, %	44.0/56.0	45.0/55.0	-
Type 2 diabetes family history, %	56.2	63.6	-
Obesity family history, %	18.7	18.1	-
Ancestry (geographic Italian region), n (%)			
Northern Italy	2 (12.5)	1 (9.1)	-
Central Italy	4 (25.0)	4 (36.4)	<0.05
Southern Italy	10 (62.5)	6 (54.5)	-
Type 1 diabetes duration, years (median and range)	6.8 (5.0–9.2)	6.5 (5.2–9.1)	-
Height, SDS	0.27 ± 0.26	0.32 ± 0.21	-
Body Mass Index (BMI), SDS	2.04 ± 0.12	2.00 ± 0.09	-
Waist circumference, SDS	2.29 ± 0.20	2.28 ± 0.27	-
Screen time (computer, TV and video) (%)			
≤2 h/day	31.2	27.2	-
2–4 h/day	37.6	36.4	-
≥4 h/day	31.2	36.4	-
Time weekly spent for exercise (%)			
≤2 h/week	25.0	27.3	-
2–4 h/week	62.5	55.4	-
≥4 h/week	12.5	17.3	-
Dietary glycaemic index	56.35 ± 2.11	50.55 ± 2.05	-
Dietary glycaemic load, units	132.25 ± 13.15	130.00 ± 15.15	-

Values are expressed as means and standard deviations (SD) for continuous variables, and data from categorical variables are shown as median and range or percentages.

**Table 2:** Clinical characteristics, glucose variability and hypoglycaemia measurements in children PGR-treated and untreated with type 1 diabetes at baseline and 3 and 6 months.

	Treated			Untreated		
Variable	Baseline	3 Months	6 Months	Baseline	3 Months	6 Months
Subjects, number (M:F)	16 (8/8)	16 (8/8)	16 (8/8)	11 (6/5)	11 (6/5)	11 (6/5)
Age, years (Median and range)	12.9 (9.5–15.8)	13.2 (9.8–16.1)	13.5 (10.0–16.4)	12.6 (9.4–15.6)	12.9 (9.7–15.9)	13.2 (10.0–16.1)
Prepubertal/pubertal ratio, %	44/56	37/63	31/69 *	45/55	55/45	36/64
Height, SDS	0.27 ± 0.26	0.28 ± 0.24	0.34 ± 0.29	0.32 ± 0.21	0.34 ± 0.23	0.37 ± 0.25
BMI, SDS	2.04 ± 0.12	1.99 ± 0.17	1.88 ± 0.16 **	2.00 ± 0.09	1.99 ± 0.15	2.01 ± 0.15 ^
Waist circumference, SDS	2.29 ± 0.20	2.23 ± 0.23	2.04 ± 0.19 **	2.28 ± 0.27	2.26 ± 0.25	2.24 ± 0.27 ^
Screen time (computer, TV and video) (%)						
≤2 h/day	31.2	37.6	43.7 *	27.2	36.4	36.4
2–4 h/day	37.6	31.2	31.2	36.4	27.3	36.4
≥4 h/day	31.2	31.2	25.1	36.4	36.4	27.3
Time weekly spent for exercise (%)						
≤2 h/week	25.0	31.2	37.6 *	27.3	36.3	36.3
2–4 h/week	62.5	62.5	56.2	55.4	45.5	45.5
≥4 h/week	12.5	6.3	6.2 *	17.3	18.2	18.2
HbA1c, % (range)	9.30 (7.4–10.2)	8.85 (7.3–10.0)	8.20 (7.0–9.5) *	9.25 (7.6–10.3)	9.15 (7.5–10.1)	9.10 (7.4–10.0) ^
Blood glucose measurements per day, n	8.1 ± 1.2	7.7 ± 1.2	7.6 ± 1.1	8.2 ± 1.2	8.0 ± 1.1	8.0 ± 1.1
Fasting BG, mmol/L	8.96 ± 1.31	8.03 ± 1.02 *	7.63 ± 0.88 **	8.89 ± 1.27	8.66 ± 1.17	8.60 ± 1.08 ^
Pre-lunch BG, mmol/L	11.42 ± 2.48	10.86 ± 2.11	9.21 ± 1.73 *	11.56 ± 2.39	11.23 ± 2.21	11.06 ± 2.00 ^
Pre-dinner BG, mmol/L	11.63 ± 2.26	11.37 ± 2.22	10.01 ± 2.07 *	11.84 ± 2.33	11.58 ± 2.28	11.76 ± 2.23 ^
Postprandial BG, mmol/L	12.09 ± 2.21	11.64 ± 2.13	10.41 ± 2.02 **	12.23 ± 2.29	12.19 ± 2.16	12.14 ± 2.19 ^
Mean insulin dose, u/kg/die	1.47 ± 0.49	1.16 ± 0.34 *	1.03 ± 0.29 **	1.45 ± 0.43	1.37 ± 0.36	1.38 ± 0.37 ^
Mean daily blood glucose, mmol/L	9.48 ± 1.57	8.78 ± 1.41	7.99 ± 1.12 **	9.29 ± 1.53	9.17 ± 1.48	9.13 ± 1.42 ^
SD, mmol/L	3.45 ± 0.57	2.99 ± 0.51 **	2.28 ± 0.43 ***	3.39 ± 0.59	3.22 ± 0.56	3.03 ± 0.51 ^^
Mean coefficient of variation, %	36.39%	34.05%	28.53% *	36.49%	35.11%	33.18%
LBGI	5.64 ± 2.33	4.42 ± 2.11 *	2.55 ± 1.87 ***	5.73 ± 2.59	5.47 ± 2.43	5.13 ± 2.27 ^^
HBGI	9.87 ± 2.79	8.83 ± 2.21 *	5.46 ± 1.91 ***	9.94 ± 2.98	9.47 ± 2.71	9.06 ± 2.56 ^^^
GRADE						
Mean score	15.0 ± 5.2	12.9 ± 4.1	10.5 ± 3.3 *	14.8 ± 5.0	14.2 ± 4.8	14.5 ± 4.5 ^
Hyperglycaemic, %	66.2 (51.5–81.0)	59.4 (48.1–73.4)	54.7 (45.4–66.1) *	65.6 (51.7–82.3)	64.4 (52.1–79.4)	64.2 (51.6–78.4)
Euglycemic, %	23.2 (15.8–33.3)	31.4 (20.4–47.8)	38.7 (33.2–53.4) **	23.0 (10.0–32.6)	24.0 (12.1–31.2)	24.3 (10.8–40.1) ^^
Hypoglycaemic, %	10.6 (0.8–27.3)	9.2 (0.6–25.4)	6.6 (0.5–22.3) *	11.4 (0.9–25.4)	11.6 (0.8–26.1)	11.5 (0.8–25.7) ^
J-index	54.16 ± 17.22	44.88 ± 13.63	34.17 ± 9.11 ***	52.09 ± 16.88	49.73 ± 14.54	47.90 ± 13.99 ^^
MODD, mmol/L	6.4 ± 2.6	5.2 ± 2.1	4.7 ± 1.6 *	6.4 ± 2.5	6.3 ± 2.4	6.2 ± 2.4 ^
ADRR °	44.91 ± 14.29	40.28 ± 12.99 *	32.75 ± 10.08 *	44.83 ± 13.76	43.99 ± 14.11	43.94 ± 13.53 ^
MAGE	7.49 ± 1.76	6.33 ± 1.02	5.57 ± 1.13 ***	7.41 ± 1.70	6.99 ± 1.62	6.93 ± 1.63 ^
Hyperglycaemia index	1.9 ± 1.0	1.6 ± 0.9	1.2 ± 0.8 *	1.9 ± 1.2	1.8 ± 1.0	1.8 ± 0.9
Hypoglycaemia index	1.8 ± 1.0	1.2 ± 0.8	0.9 ± 0.7 *	1.8 ± 1.1	1.9 ± 1.1	1.7 ± 0.9 ^
Index of Glycaemic Control	3.7 ± 1.0	2.8 ± 0.9	2.1 ± 0.8 **	3.6 ± 1.2	3.3 ± 1.1	3.4 ± 0.9 ^^^
Patients experienced morning hypoglycaemia, %	12.5	6.3 *	6.3 *	9.1	9.1	9.1
Patients experienced nocturnal hypoglycaemia, %	18.7	12.5	12.5	27.3	27.3	27.3 ^^^
Morning hypoglycaemia episodes per month per patient, n	3.9 ± 1.1	3.3 ± 1.0	2.9 ± 1.0 *	4.0 ± 1.2	3.9 ± 1.1	3.8 ± 1.2 ^
Nocturnal hypoglycaemia episodes per month per patient, n	6.7 ± 3.0	5.5 ± 2.7	4.1 ± 2.5 *	6.6 ± 3.3	6.3 ± 3.2	6.1 ± 3.0 ^
Triglycerides, mmol/L	1.81 ± 0.39	1.65 ± 0.34	1.51 ± 0.31 *	1.83 ± 0.41	1.80 ± 0.38	1.74 ± 0.37
Total cholesterol, mmol/L	5.41 ± 0.64	5.29 ± 0.47	4.81 ± 0.41 **	5.48 ± 0.69	5.37 ± 0.61	5.33 ± 0.60 ^
HDL-cholesterol, mmol/L	0.86 ± 0.13	0.93 ± 0.14	1.01 ± 0.13 **	0.92 ± 0.14	0.94 ± 0.15	0.90 ± 0.14 ^
LDL-cholesterol, mmol/L	3.73 ± 0.52	3.61 ± 0.50 *	3.12 ± 0.37 ***	3.73 ± 0.53	3.62 ± 0.51	3.64 ± 0.52 ^^
Systolic BP, SDS	2.11 ± 0.56	1.83 ± 0.62	1.63 ± 0.60 *	2.14 ± 0.63	2.07 ± 0.56	2.11 ± 0.57 ^
Diastolic BP, SDS	2.17 ± 0.59	1.99 ± 0.60	1.71 ± 0.58 *	2.09 ± 0.57	2.11 ± 0.59	2.08 ± 0.58

Data are reported as mean ± SD or geometric mean (range) values. SDS, standard deviation score; BMI, body mass index; BP, blood pressure; HbA1c, glycated haemoglobin; BG, blood glucose; ADRRs, average daily risk range using self-monitoring of blood glucose; GRADE, glycaemic risk assessment diabetes equation; LBGI, Low Blood Glucose Index; MAGE, mean amplitude of glycaemic excursions; MBG, mean blood glucose; HDL, high-density lipoprotein; LDL, low-density lipoprotein; IU, international unit. Paired Student’s *t*-test: * *p* < 0.05 vs. baseline; ** *p* < 0.005 vs. baseline; *** *p* < 0.0005 vs. baseline; ^ *p* < 0.05 vs. treated group; ^^ *p* < 0.005 vs. treated group; ^^^ *p* < 0.0005 vs. treated group. ° ADRR was computable on 24 patients datasets only.

## Data Availability

The datasets used and/or analyzed during the current study are available from the corresponding author on reasonable request.

## Supplementary Materials

The following are available online at https://www.mdpi.com/article/10.3390/nu13103517/s1, Supplementary file: Patients and Methods Section.
