# Supplementary material for: Retrospective Evaluation on the Use of a New Polysaccharide Complex in Managing Paediatric Type 1 Diabetes with Metabolic Syndrome (MetS)
Source: Nutrients. 2021 Oct 7;13(10):3517. doi: 10.3390/nu13103517 (PMC8540288; doi:10.3390/nu13103517)
Supplement: Supplementary file 1 [file nutrients-13-03517-s001.zip › nutrients-1367922-supplementary.pdf]

Patients and Methods Section, Supplementary Materials

The ideal BG level for the M value in this study was set at 5.5 mmol/L, with 4.44 mmol/L as the lower limit and 7.77 as the upper limit of the target range. GV was defined as the degree to which a patient's BG fluctuated between high and low levels [34]. As HbA1c estimates the average glycaemic exposure over 2 to 3 months, the same HbA1c value may be experienced by patients who had significantly different BG fluctuations [35]. In addition, the frequencies of hypoglycaemic and hyperglycaemic events (glucose meter readout of <3.88 and >10 mmol/L respectively) were assessed using standard patient diaries.

Hypoglycaemic events were defined on the basis of symptoms consistent with hypoglycaemia associated with a BG level of 3.88 mmol/L or followed by a prompt recovery after oral carbohydrate, and were classed as severe when the hypoglycaemic event required the administration of intravenous glucose or subcutaneous glucagon and the assistance of another person [36]. They were categorised as follows: frequent: two or more times per week; nocturnal: between bedtime and 06:00 a.m.; morning: between 06:00 a.m. and breakfast [36]. The number of events (Δhypoglycaemia) was also monitored, and calculated as the difference in the number of episodes of glycaemia <70 mg/dL (3.88 mmol/L) per week per patient, as well as the relative percentage of change.

As reported above, hyperglycaemia was defined as BG level >10.0 mmol/L. However, the calculation of the hyperglycaemic index was also based on SMBG and was defined as the area under the glucose curve above the normal range divided by the total observation period [36].

We also calculated the mean blood glucose, which is thought to provide better insight into glycaemic variability, by the formula: $\frac{\sum_{t=t_1}^{t_k} GV_t}{k}$ where k is the number of glucose values (GVs) in a given individual. In addition to the mean BG, insufficient for evaluating blood glucose oscillations, the SD was also calculated [37], as well as the percent coefficient of variation (%CV = 100 × SD/mean) as the ratio of SD to mean. This parameter describes the magnitude sample values and

the variation within them and enables standardised comparisons between subjects with different levels of mean glycaemia [38].

For the assessment of the glycaemic variability, other parameters were also calculated [28, 39-42]. LBGI and HBGI were also calculated. These were defined as indices based on a nonlinear transformation of the BG scale, applying symmetry to the distribution of BG readings for a subject. These are non-negative numbers, their sum ranging from 0 to 100. The LBGI indicates risk for hypoglycaemic excursions, whereas HBGI indicates risk for hyperglycaemic excursions. Empirically derived risk categories were as follows: LBGI: Minimal (LBGI ≤1.1), Low (1.1 <LBGI ≤2.5), Moderate (2.5 < LBGI ≤5) and High (LBGI >5.0);28 HBGI: Low (HBGI ≤4.5), Moderate (4.5 <HBGI ≤9.0) and High (HBGI >9.0) [37].

Low or High Blood Glucose (BG) Index (LBGI and HBGI: provide early risk indicators for hypoglycaemia and hyperglycaemia),

J-index, as proposed by Wójcicki [38], was also used for the assessment of glycaemic variability and was calculated by the formula: $J = 0.324 \times (MBG + SD)^2$ where mean BG (MBG) is the MBG level measured in mmol/L and SD is the SD of glucose levels.

MAGE was calculated to take into account the blood glucose peaks and nadirs encountered during a day, rather than just mean glucose values, so as to give more weight to major variations and less weight to minor ones. MAGE was calculated according to the formula: $\sum \frac{\lambda}{\chi}$ if $\lambda > y$ where λ is the difference from peak to nadir, χ is the number of valid observations and y is 1 SD of mean glucose in a 24-h period [39].

To correct the skewed distribution of glucose readings, ADRR was also calculated [26] from 2-4 weeks of routine self-monitoring of BG readings with a frequency of three or more readings per day, converting the resulting values into risk values using the formula r(BG) = f(BG). ADRR was thus calculated using the formula: $ADRR = \frac{1}{M} \sum_{i=1}^{M} LR^i + HR^i$ where LR^i and HR^i represent the maximums of, respectively, the left and the right branch of the resulting parabola of the formula

r(BG) = f(BG). ADRR <20 represents a low risk, 20–40 corresponds to a moderate risk and values >40 indicate a high risk for BG excursions.

Inter-day blood glucose variation was calculated using the MODD, i.e. the mean absolute value of the difference between glucose values taken at the same time on two consecutive days. Higher MODD values may indicate poorer glycaemic control across multiple days and/or a more irregular daily schedule.

Finally, GRADE was also calculated. GRADE is a family of methods attempting to measure the quality of glycaemic control, and not simply glycaemic variability. It is the percentage of time spent by glucose value in given % ranges corresponding to hypoglycaemia ($\%GRADE_{hypoglycaemia}$), euglycaemia ($\%GRADE_{euglycaemia}$) and hyperglycaemia ($\%GRADE_{hyperglycaemia}$) [40].
